# A 24 hour neuroimaging protocol to track solute transport from blood to the CSF as a surrogate for protein clearance

**DOI:** 10.1002/alz70856_102525

**Published:** 2025-12-25

**Authors:** Swati Rane Levendovszky, Briana R Meyer, Jeffrey J Iliff

**Affiliations:** ^1^ University of Washington School of Medicine, Seattle, WA, USA; ^2^ University of Washington, Seattle, WA, USA

## Abstract

**Background:**

Neurofluids play a key role in clearing amyloid and tau proteins from the brain. MRI studies using intrathecal gadolinium‐based contrast agents (GBCA) show transport from the cerebrospinal fluid (CSF) into brain tissue. Additionally, studies using intravenous (IV) GBCA demonstrate blood‐to‐CSF transfer, though these studies often include limited time points, when vascular GBCA concentration is high, making it hard to parse parenchymal or CSF concentration. We performed hourly MRI scans over 24 hours after IV GBCA to detect CSF contrast transport, aiming to track protein movement through the brain.

**Method:**

Seven healthy adults (ages 23‐57, 2 female) underwent 24 MRI scans, starting with a baseline, followed by scans at 20 minutes and then hourly from 1 to 22 hours post‐IV GBCA (Gadobutrol, 0.1 mmol/kg). Imaging included 3D‐QALAS for T1/T2 mapping, a heavy T2‐weighted FLAIR sequence, and a 3D Black Blood sequence. T1‐w images from the QALAS provided tissue masks for gray matter (GM), white matter (WM), subarachnoid space (SAS), and the lateral ventricles (LV). Manual regions included the sagittal sinus (SS), optic nerve (O), trigeminal nerve (T), choroid plexus (CP), and arachnoid cuff exit (ACE) points. Signal intensity changes were noted for all time points in all regions. GBCA concentration was calculated using T1 mapping data.

**Result:**

In the CSF, GBCA concentration was 0.007 mM in the SAS and 0.0007 mM the LV at 20 mins, and then quickly settled to 0.002 and 0.001 mM until 22 hours. The SAS had a higher GBCA concentration than LV, suggesting a direct blood‐to‐CSF exchange near the SAS. This could be explained by contrast leakage around ACE points, visible on T2w FLAIR images. Additionally, the Black Blood sequence showed sustained GBCA in the parasagittal dura. GBCA was retained the longest in the optic nerve (∼19 hours), while it dissipated fastest around ACE points. No enhancement was observed in the parenchyma. In the CP, the concentration was 0.02 mM at 20 mins but was negligible by 4 hours.

**Conclusion:**

IV‐GBCA can track contrast transport from the vascular to the CSF compartment. GBCA transfers to the ventricles and SAS through multiple mechanisms, with varying transport rates across different nerves.

